# CXCL9 as a key biomarker of vitiligo activity and prediction of the success of cultured melanocyte transplantation

**DOI:** 10.1038/s41598-021-97296-2

**Published:** 2021-09-14

**Authors:** Fuquan Lin, Wenting Hu, Wen Xu, Miaoni Zhou, Ai‑E. Xu

**Affiliations:** Department of Dermatology, Hangzhou Third People’s Hospital, Affiliated Hangzhou Dermatology Hospital, Zhejiang University School of Medicine, Hangzhou, China

**Keywords:** Immunology, Biomarkers, Diseases, Medical research

## Abstract

This study aimed to investigate the potential biomarkers of vitiligo by evaluating the disease activity and curative effect of autologous cultured pure melanocyte transplantation (CMT) on patients. Altogether, 36patients with stable vitiligo were treated with CMT. Blister fluid samples were collected from patients with stable vitiligo. Patients with active vitiligo were matched with healthy controls. The chemokine levels in the serum and blister fluid samples were measured using Luminex. The curative effect on patients with stable vitiligo was evaluated 6 months after treatment. Treatment responses were defined according to the extent of repigmentation as effective (if 50% or more repigmentation was achieved) or ineffective (if less than 50% or worse repigmentation was achieved). Patients received re-transplantation if the initial treatment was ineffective. The levels of C-X-C motif chemokine ligand (CXCL)9 and CXCL10 in blister fluid samples were significantly lower in stable patients than in active participants. Receiver operating characteristic analysis revealed that the levels of CXCL9 and CXCL10 were sensitive and specific in diagnosing active vitiligo. Further, 65.6% (21/32) of patients who received CMT had effective treatment responses. The high CXCL9 level in the blister fluid was a significant predictor of ineffective treatment responses. The treatment response was significantly enhanced after treatment. Four patients with ineffective treatment responses received anti-inflammatory treatment and re-transplantation. The CXCL9 and CXCL10 levels in the blister fluid were related to the presence of active vitiligo. Also, the CXCL9 level was a predictor of the effectiveness of CMT in treating vitiligo.

## Introduction

Vitiligo is a common pigmentary skin disease that affects 0.1–2% of individuals among various populations, and only some patients with vitiligo would respond to diverse medical treatments^1–5^. In refractory and stable vitiligo, a surgical procedure is required to improve the repigmentation rate^6^. In our previous studies, the application of autologous cultured pure melanocyte transplantation (CMT) in stable vitiligo achieved favorable outcomes^7–9^.

Vitiligo is characterized by the loss of functional melanocytes. It is considered that the pathogenic mechanisms are different between segmental and nonsegmental vitiligo due to their different clinical patterns. However, recent data indicate the overlapping inflammatory pathogenesis of segmental and nonsegmental vitiligo, both of which are closely related to T-cell immunity. Increasing evidence has shown that vitiligo is induced by the activated melanocyte antigen-specific CD8^+^ T cells that drive cytotoxicity and disease pathogenesis^10–13^. Recruitment of auto-reactive CD8^+^ T cells to melanocytes is mediated by interferon γ (IFN-γ) via IFN-γ-induced chemokines, such as C-X-C motif chemokine ligand (CXCL) 9 and CXCL10^14–18^.CXCL9 and CXCL10 are the Th1 chemokines induced by IFN-γ in various cell types, such as neutrophils, lymphocytes, and endothelial cells as well as fibroblasts and other epithelial cells ^19,20^. BothCXCL10 and CXCL9 can directly bind to the specific receptor, chemokine (C-X-C motif) receptor (CXCR)3, to subsequently regulate immune responses by recruiting and activating T cells, monocytes, and also natural killer cells ^19,20^. These signaling pathways are regulated by Janus kinase (JAK) 1 and 2^21,22^. CXCL9 and CXCL10 have been validated as the biomarkers of vitiligo activity^18,23^. Their levels in the skin and blood of patients with vitiligo are higher than those in healthy controls, and the levels in patients with active vitiligo are higher than those in patients with stable vitiligo^14,15,18^.

According to a newly published study, a reduction in the serum CXCL10 level predicts better treatment outcomes in patients with vitiligo receiving ruxolitinib treatment^24,25^. Rashighiet al. showed that CXCL10 was essential for the progression and maintenance of depigmentation in a mouse model of vitiligo^15^. Ferrariet al. found that the level of circulating CXCL10 increased in nonsegmental vitiligo, regardless of the presence of autoimmune thyroiditis^19^. These results suggested that Th1 immune response was essential for the immune-pathogenesis of vitiligo^19^.

Thus, it was hypothesized in this study that chemokines CXCL9 and CXCL10might be the potential biomarkers for predicting treatment response in patients with vitiligo undergoing CMT. A prospective study was performed to observe the different chemokine levels in patients with vitiligo treated with CMT (Table 1).

**Table 1:** Demographic and clinical data of stable vitiligo group (treatment group), active vitiligo group, and control group.

	L-stable vitiligo	L-active vitiligo	NL-stable vitiligo	Health control	*P* value
Name of cases	32	13	10	10	–
Age	36.7 ± 10.2	37.5 ± 9.9	35.5 ± 7.9	28.6 ± 5.7	> 0.05
Gender(male/female)	18/14	7/6	6/4	5/5	> 0.05
Type pf vitiligo (localized/general/segmental)	26/25	8/3/2	–	–	> 0.05
Period of stability (month)	19.2 ± 10.0	–	–	–	–
Location head neck/trunk	14/18	0/13	0/10	–	–

## Results

### Baseline clinical characteristics of patients

**Figure 1:**
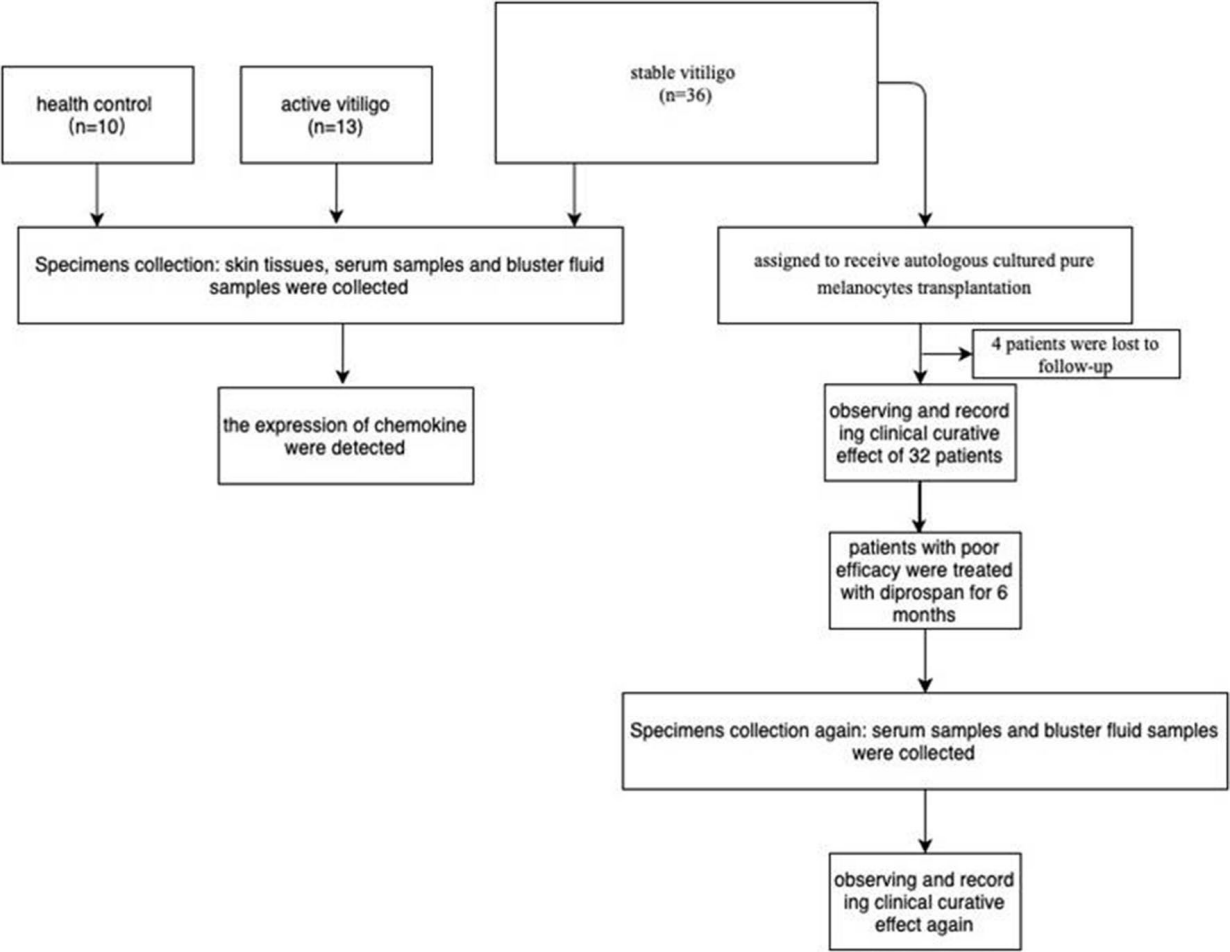
Flow chart of the study.

Of all the36 patients with stable vitiligo, 32 (including14 female and 18male;mean age, 36.7 ± 10.24 years) completed the 6- to 12-month follow-up, while 4 dropped out at the 3-month follow-up (Fig. 1); the mean stable time was 19.2 months. The mean (standard deviation) age at the onset of active vitiligo was 37.5 ± 9.86 years, and serum and blister fluid samples were collected from 13 of these patients, including 6 female and 7 male patients, with the mean age of 35.5 ± 7.89 years. In addition, blister fluid samples were collected from 10 control participants with nonlesional stable vitiligo, and serum samples were collected from 10 healthy controls. No statistically significant difference in age or sex was found among different groups. Stable vitiligo was mainly observed in the face, neck, and trunk, and active vitiligo mainly occurred in the trunk. Further, no significant difference was found in the proportion of patients with mixed types of vitiligo (localized/general/segmental).

### Increased chemokine levels in the serum and blister fluid samples of patients with vitiligo

**Figure 2:**
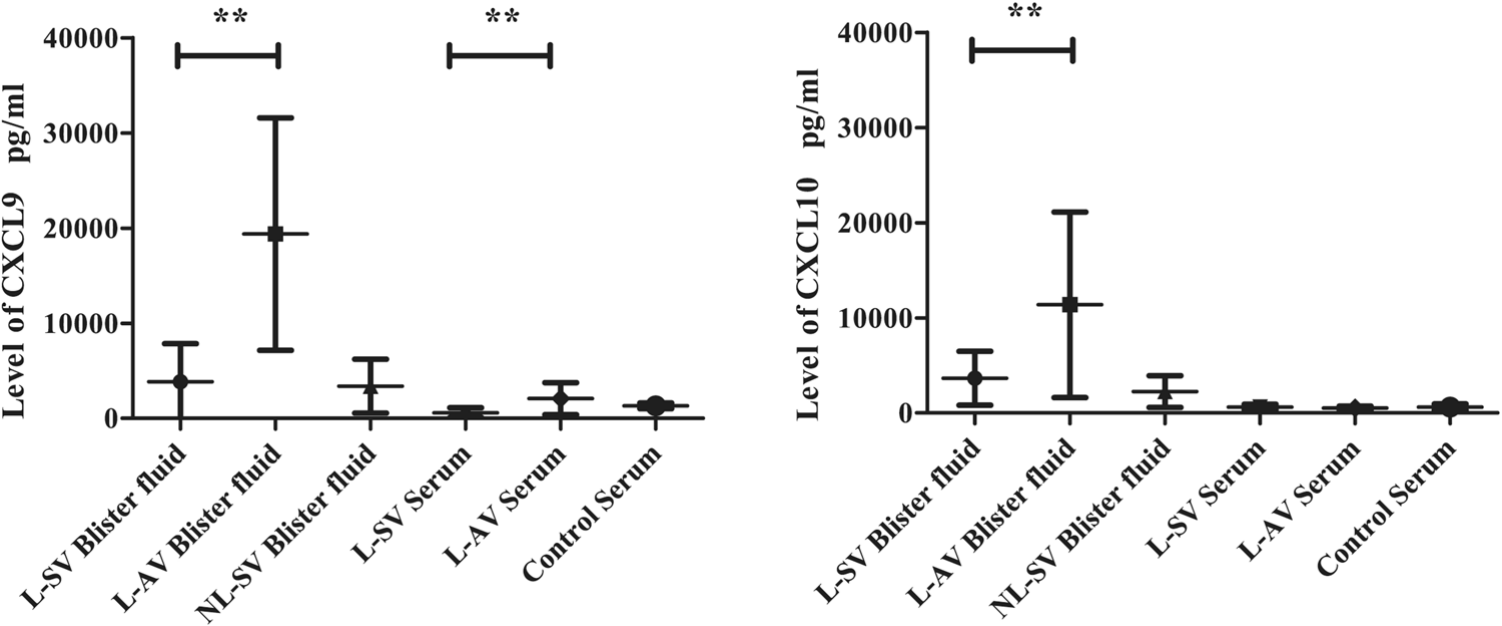
The mean levels of CXCL9 and CXCL10 in different groups. *P < 0.05, **P < 0.01.

This study detected the levels of innate chemokines in both serum and blister fluid samples. Together, these results in Table 2 showed that the levels of CXCL9and CXCL10 increased in both patients with stable vitiligo and patients with active vitiligo (*P* < 0.01).As shown in Fig. 2, the expression of CXCL9 and CXCL10in the blister fluid was significantly higher in patients with active vitiligo than in patients with stable vitiligo and controls(*P* < 0.01).However, no statistically significant differences were found in the serum level of CXCL10 among the three groups(*P* > 0.05) after Unpaired t-test. The serum expression level of CXCL9 was significantly higher in patients with active vitiligo than in patients with stable vitiligo(*P* < 0.01), but the difference was not significant compared with the control group(*P* > 0.05), and the level in the control group was higher than that in patients with stable vitiligo.

**Table 2:** The mean values of blister fluid and serum CXCL9/CXCL10 levels in vitiligo patients and controls.

	Level of CXCL9 (pg/ml)					Level of CXCL10 (pg/ml)				
	L-SV	L-AV	NL-SV	Control	P value	L-SV	L-AV	NL-SV	Control	P value
Blisterfluid	3856 ± 3987	19,370 ± 12,230	3388 ± 2828	–	0.000	3659 ± 2828	11,377 ± 9746	2257 ± 1676	–	0.000
Serum	599 ± 532	2080 ± 1683	–	1321 ± 290	0.002	652 ± 290	512 ± 203	–	626 ± 330	0.26

L-SV stands for stable vitiligo group, L-AV represents active vitiligo group, NL-SV indicates non-lesional stable vitiligo group, and the control group is the healthy control group. P-value indicates statistical significance between L-SV and active vitiligo patients statistical difference.

**Figure 3:**
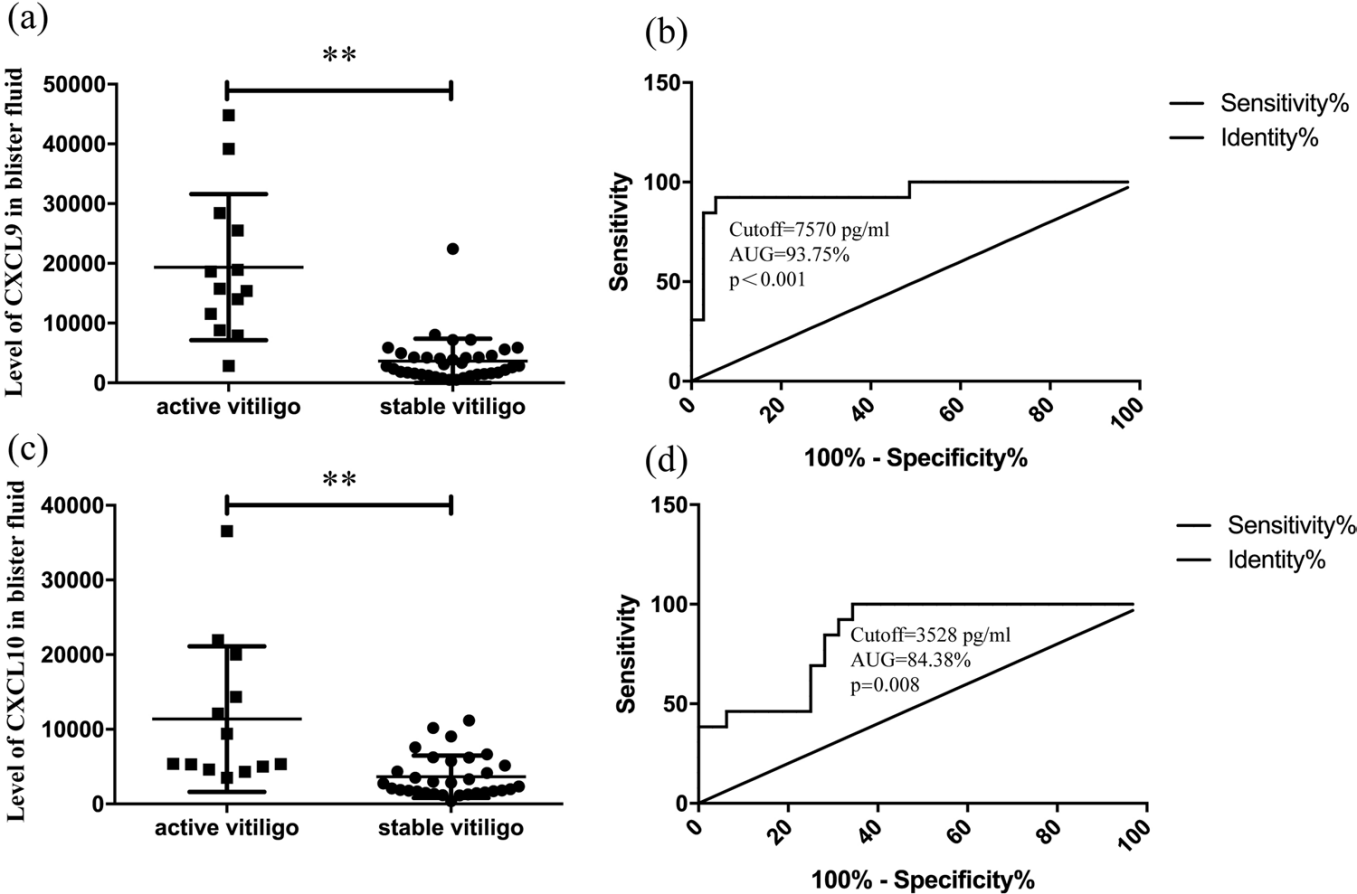
(**a**, **c**) The levels of CXCL9 and CXCL10 in blister fluid. (**b**, **d**) ROC curves plotted to distinguish the cut-off values of CXCL9 andCXCL10 among active patients and stable vitiligo patients. *P < 0.05, **P < 0.01.

Later, the cutoff value of the CXCL9level in the blister fluid with respect to the incidence of active vitiligo was calculated. Receiver operating characteristic (ROC) analysis showed that the cutoff value of the CXCL9 level in the blister fluid was 7570 pg/mL (area under the curve = 0.9375; sensitivity = 92.3%; and specificity = 93.8%). Moreover, Fig. 3 shows that the cutoff value of the CXCL10 level in the blister fluid was 3528 pg/mL (area under the curve = 0.8438; sensitivity = 100%; and specificity = 65.63%).

### Clinical effects

**Figure 4:**
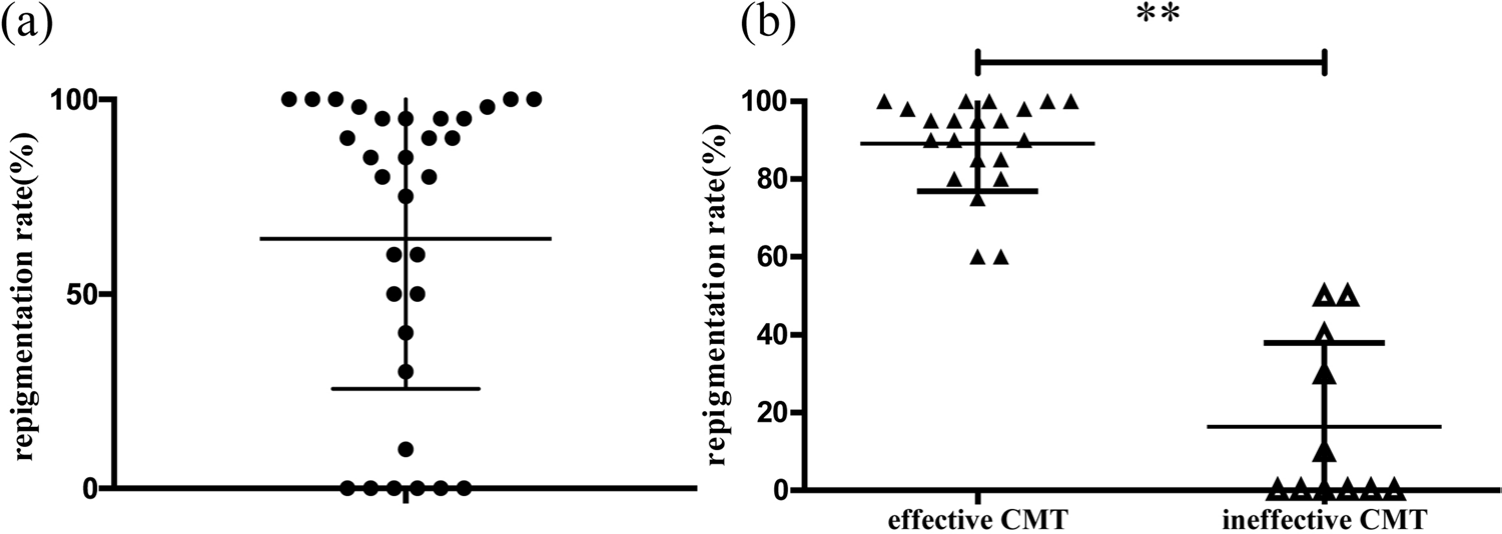
(**a**)There pigmentation rate in stable vitiligo patients (n = 32). (**b**) Statistics of differences between groups of different CMT effects. *P < 0.05, **P < 0.01.

Patients with large-area stable vitiligo received CMT surgical treatment. The mean repigmentation rate of CMTwas64.1% (P25, P75:37.5%, 95%).Further, 11 patients showed ineffective treatment responses (< 50%) (Fig. 4a). The mean repigmentation rate of ineffective CMT group (16.36%) was much lower than effective CMT group (89.09%) after Mann–Whitney U-test (P < 0.01) (Fig. 4b).

### Analysis of factors that might affect the therapeutic effects

**Figure 5:**
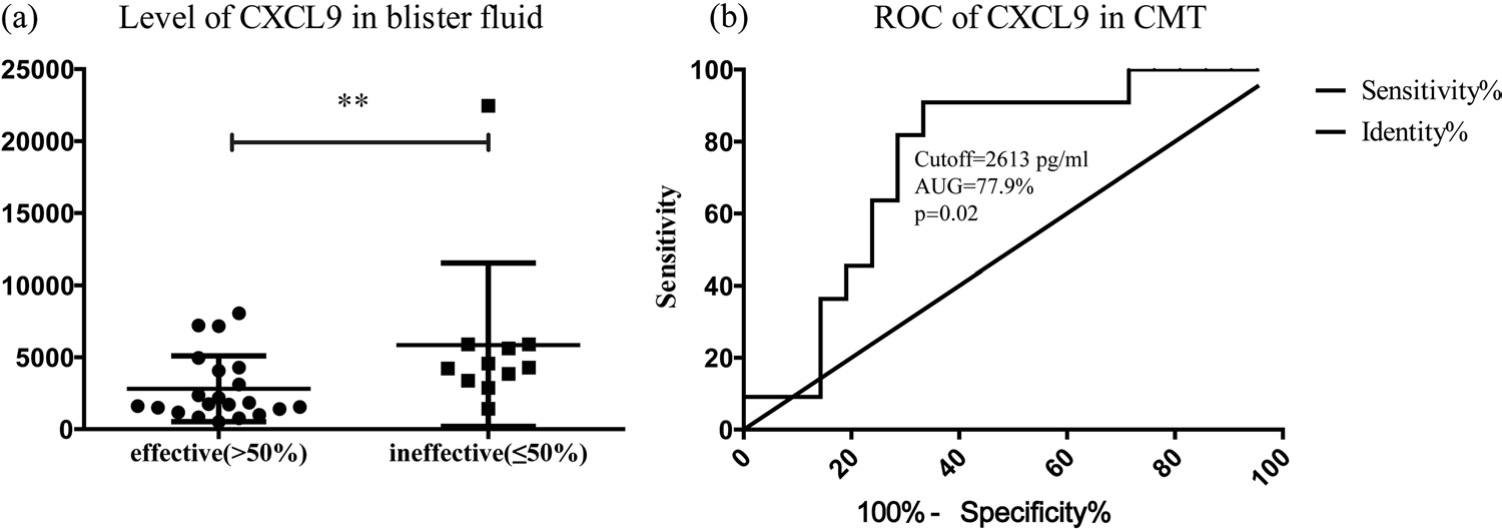
(**a**) The CXCL9 levels in blister fluid of patients with different treatment responses.(**b**) ROC curve plotted to distinguish the cut-off value of CXCL9 levels among patients with different treatment responses. *P < 0.05, **P < 0.01.

Multivariate analysis was carried out in 11 patients with ineffective treatment responses to CMT. The results showed that the differences in the type, location, stabilization time, and duration of vitiligo were not statistically significant, but the CXCL9 level in the blister fluid of 6 out of 11 patients was higher than 5000 pg/mL, close to the cutoff value of active vitiligo. The univariate analysis revealed that high expression of CXCL9 was a significant independent predictor of the ineffective treatment response to CMT in patients with vitiligo. Meanwhile, the cutoff value of the CXCL9 level with respect to the ineffective treatment response rate was calculated. The ROC analysis showed that the cutoff value of the CXCL9 level in blister fluid samples was 2613 pg/mL (area under the curve = 0.779; sensitivity = 90.9%; and specificity = 66.7%) (Fig. 5).

### Expression of chemokines and clinical effects in re-transplant patients

**Figure 6:**
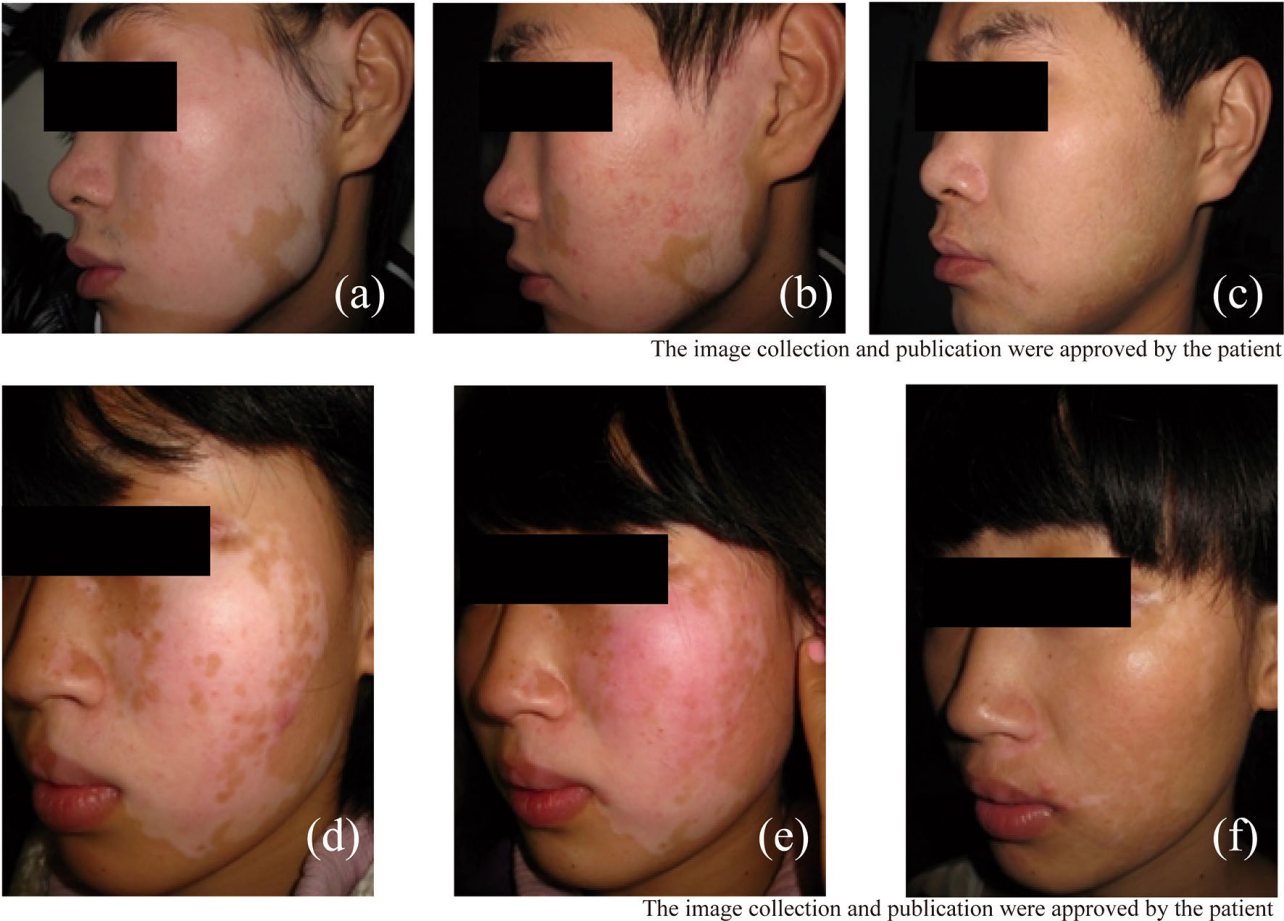
Vitiligo lesion in a 37-year-old patient (**a**) before and (**b**) 6 months after the transplantation of CMT. A0% repigmentation was achieved in the first transplantation. After treatment, a 98% repigmentation rate was achieved (**c**). Vitiligo lesion in a 22-year-old patient (**d**) before and (**e**) 6 months after the transplantation of CMT. A 0% repigmentation was achieved in the first transplantation. After treatment, a 95% repigmentation rate was attained in the re-transplantation (**f**).

Of six patients who achieved the zero repigmentation rate after CMT, we selected four whose CXCL9 levels were relatively higher than those in other patients (5000 pg/mL) to receive diprospan compound betamethasone injection. The CXCL9 level in the blister fluid at the donor site was detected in 6 months to 1 year after injections. In the re-transplant patients, the CXCL9 level in the blister fluid showed a statistically significant difference before and after CMT treatment. Also, compared with primary transplantation (Fig. 6b,e), the pigmentation rate of re-transplantation increased dramatically (Fig. 6c,f). More than 95% of the area could be cured and recovered after successful CMT treatment than before treatment (Fig. 6a,d).

## Discussion

Transplantation of autologous cultured pure melanocytes is a well-established procedure used to treat stable vitiligo^8,26–29^. However, defining the concept of stability in the setting of vitiligo is relatively difficult. Vitiligo has an unpredictable clinical course, including the periods of disease stability and disease flares^2,3,5^. Establishing a definitive diagnosis of active vitiligo or stable vitiligo is challenging, which depends on the clinical manifestation of patients^16,17,30^. Biomarker analysis maybe useful for subsequent patients over time, which can even predict future disease progression to tailor individualized treatment^18^.

As suggested by a previous study, both CXCL9 and CXCL10 play functional roles in the mouse model of vitiligo^15^. Although these two chemokines are expressed in active vitiligo, they are produced by different cell types with different kinetics during the progression of vitiligo. Of them, CXCL9 is expressed the earliest in murine vitiligo lesions, and may be a more specific marker of disease activity^31^.

Our results were consistent with the previous findings^14–16,18^. This study found that the levels of CXCL9 and CXCL10 in the blister fluid were significantly higher in patients with active vitiligo than in patients with stable vitiligo. Similar small-scale studies showed that the CXCL9 protein in the blister fluid achieved greater sensitivity and specificity in active vitiligo and might be an effective early marker of treatment response^17^. No significant difference was found in the serum CXCL9 level between patients with active vitiligo and stable vitiligo. CXCL9 is presumably more sensitive than CXCL10. CXCL9 and CXCL10 can detect visible differences in serum and blister fluid samples, and their levels in the blister fluid may be the best indicators. Of these, CXCL9 achieved better accuracy, with no difference compared with CXCL10 alone in detecting serum samples. Typically, the accuracy of the blister fluid was only 87% in active vitiligo, while it was less than 50% in vitiligo in the treatment group; therefore, CXCL9 might serve as a good marker. Also, CXCL9 expression was found to be related to the treatment response, which might also be used to predict the response to CMT treatment in patients with vitiligo. In patients treated with CMT, the cutoff level of CXCL9 in the blister fluid was2613 pg/mL. It suggested that the high concentration of CXCL9 might indicate disease activity and local inflammation, which might prevent the survival of transplanted melanocytes and reduce the treatment effectiveness.

The four patients with ineffective treatment response after the primary transplantation received follow-up treatment. Over the 6-month follow-up period, diffuse repigmentation was found in the transplanted areas, which disappeared after several months. This phenomenon reflected the progress of the transplanted areas. The CXCL9 level in these patients during the primary transplantation was higher than2613pg/mL. We assumed that melanocyte destruction was caused byCXCL9 and its receptor CXCR3, which triggered T-cell proliferation and adaptive response by lymphocytes^32,33^.

After the application of diprospan (compound betamethasone injection^34,35^, 1 mL, once a month for 3 months), the CXCL9 level in the blister fluid was significantly lower than that before, and the curative effect was significantly improved. This result indicated that the CXCL9 expression level was directly and negatively related to the efficacy of CMT. Thus, we concluded that patients with a high CXCL9levelin the blister fluid might not be good candidates for CMT. The relatively small sample size was the limitation of this clinical observation. Strassner et al. presented a reliable validation cohort of multiple patients to validate CXCL9/10 as the biomarkers of vitiligo disease activity, While this study only followed one patient define the treatment response^17^. It is difficult to achieve the monitoring and guiding significance of the therapeutic effect. We conducted an ROC analysis of 32 cases of CMT treatment. The systemic treatment of patients with poor efficacy and a high CXCL9 level could significantly stabilize the disease and improve the efficacy.

Skin biopsy is considered too invasive for most patients for biomarker purposes, while negative pressure suction is less invasive^36,37^. Therefore, monitoring the chemokine levels has a certain clinical value and may be used in the clinical diagnosis of stable vitiligo. It can also be used to check whether the lesion is suitable for transplantation.

## Conclusions

In conclusion, the activity of vitiligo has a positive correlation with the levels of CXCL9 and CXCL10 in the blister fluid. Moreover, the CXCL9 level in the blister fluid before treatment may be used as a predictive factor of the therapeutic effect of CMT. We propose that managing the CXCL9 level in the blister fluid is the most reliable indicator to guide clinical therapy and choose the timing of transplantation for patients with vitiligo.

## Methods

### Study design and participants

Our study protocol complied with the Declaration of Helsinki, and was approved by the Ethics Committee of the Third People’s Hospital of Hangzhou. As shown in Fig. 1, 36 patients with stable vitiligo (SV) were treated with CMT at the hospital from January 2016 to July 2018 (Table 1). The treated lesions were stable for 6 months or longer. Serum and blister fluid samples were collected from blood and the auto-donor site. Patients with recent onset of new lesions, objective clinical signs of activity, especially for confetti-like macules or trichrome lesions, and higher vitiligo Area Scoring Index (VASI) scores compared with the baseline VASI scores were recruited as the active subgroup. Meanwhile, subjects with stable disease or no recent change in lesions and no objective clinical sign of disease activity (see above) were selected based on patient self-reports. All patients agreed with the publication of identifying information/images in an online open-access publication.

### Serum chemokine test by Luminex assay

Peripheral serum samples were collected from 36 patients with stable vitiligo, 13 patients with active vitiligo and 10 health controls. Thereafter, the serum levels of CXCL9 and CXCL10 were examined using the Human Chemokine Panel Kits (Millipore, Bedford, MA, USA) with Luminex 200 (Millipore). The minimum detection limits were 5 pg/ml for CXCL10 and 4 pg/ml for CXCL9.

### Blister fluid chemokines measured by Luminex assay

Blister fluid samples were collected from the lesions of 36 vitiligo patients (L-SV), 13 active vitiligo patients (L-AV), and 10 the non-lesional stable vitiligo (NL-SV) controls were collected by negative pressure suction (Fig. 1). First, the lesion was anesthetized with 5% lidocaine ointment for 2 h. Then, the blister fluid was induced by press suction blister (negative pressure, 0.05 kPa) and collected via a 1-mL syringe. It took1.5 h to develop the blister, which occasionally had a little blood. Finally, the blister fluid was stored in tubes at − 80 °C. The protein levels of CXCL9 and CXCL10 in the blister fluid were measured using Luminex 200 (Millipore). The minimum detection limits and approach were the same as previously described.

### Cell culture and transplantation

The cells were separated from the epidermal sheet under a stereo-microscope. The cell suspension was centrifuged, resuspended with CHMM1 medium (F12 medium with 30 ng/mL bFGF, 30 μg/mL IBMX, 30 ng/mLGM-CSF, 100 ng/mLα-MSH ,50 μg/mL gentamicin and 10% fetalbovine serum) and seeded into a culture flask. Same as we described earlier, alidocaine cream was applied 2–4 h before transplantation. The recipient areas were then cleaned with 70% alcohol and treated with ultra-pulse CO_2_ laser (pulse rate 30–50 Hz at an energy level of 225 mJ/pulse) to remove the epidermis. The melanocyte suspension was applied to the laser-denuded area at the density of 600–1000 cells per mm^2^. The transplant area was next covered with Vaseline gauze, followed by gauze soaked with F12 medium, and finally secured with gauze and surgical tape. Tapes were removed 10 days later.

### Evaluation of results

Repigmentation of the recipient area was evaluated at 6 months after transplantation, and the repigmentation area was measured. Different treatment responses were categorized according to the extent of repigmentation as effective (50% or more repigmentation) and ineffective (< 50% or worse).

### Follow-up treatment of re-transplant for ineffective patients

After obtaining informed consents, 4 patients who failed to have effective treatment response to CMT received intramuscular injection of diprospan once a month for 3 months. Then, they were treated with CMT again, and serum and blister fluid samples were collected again during transplantation. Repigmentation of the recipient area was evaluated again at 6 months after re-transplantation.

### Statistical analysis

Data were expressed as median and inter quartile range. Perform descriptive statistics on all data, calculate the homogeneity of variance and normal distribution between the two groups. Differences between groups were analyzed by Unpaired t-test, Mann–Whitney U-test and Kruskal–Wallis test based on descriptive statistics. All analyses were performed with SPSS version 20.0 software (IBM, Armonk, NY). *P* < 0.05 (two-tailed) was considered statistically significant. descriptive statistics.

### Informed consent

Informed consent was obtained from all individual participants included in the study. Additional informed consent was obtained from two participants for whom identifying information is included in this article.

## References

[1] Ezzedine K, Lim HW, Suzuki T, Katayama I, Hamzavi I, Lan CC (2012). Revised classification/nomenclature of vitiligo and related issues: the vitiligo global issues consensus conference. Pigment Cell Melanoma Res..

[2] Abdel-Malek ZA, Jordan C, Ho T, Upadhyay PR, Fleischer A, Hamzavi I (2020). The enigma and challenges of vitiligo pathophysiology and treatment. Pigment Cell Melanoma Res..

[3] Abu Tahir M, Pramod K, Ansari SH, Ali J (2010). Current remedies for vitiligo. Autoimmun. Rev..

[4] Grimes PE (2005). New insights and new therapies in vitiligo. JAMA.

[5] Nahhas AF, Braunberger TL, Hamzavi IH (2019). Update on the management of vitiligo. Skin Ther. Lett..

[6] Taieb A, Alomar A, Bohm M, Dell'anna ML, De Pase A, Eleftheriadou V (2013). Guidelines for the management of vitiligo: the European Dermatology Forum consensus. Br. J. Dermatol..

[7] Hong WS, Hu DN, Qian GP, McCormick SA, Xu AE (2011). Ratio of size of recipient and donor areas in treatment of vitiligo by autologous cultured melanocyte transplantation. Br. J. Dermatol..

[8] Hong WS, Hu DN, Qian GP, McCormick SA, Xu AE (2011). Treatment of vitiligo in children and adolescents by autologous cultured pure melanocytes transplantation with comparison of efficacy to results in adults. J. Eur. Acad. Dermatol. Venereol. JEADV..

[9] Zhou MN, Zhang ZQ, Wu JL, Lin FQ, Fu LF, Wang SQ (2013). Dermal mesenchymal stem cells (DMSCs) inhibit skin-homing CD8+ T cell activity, a determining factor of vitiligo patients' autologous melanocytes transplantation efficiency. PLoS ONE.

[10] van den Boorn JG, Konijnenberg D, Dellemijn TA, van der Veen JP, Bos JD, Melief CJ (2009). Autoimmune destruction of skin melanocytes by perilesional T cells from vitiligo patients. J. Invest. Dermatol..

[11] Riding RL, Harris JE (2019). The role of memory CD8(+) T cells in vitiligo. J. Immunol..

[12] Oyarbide-Valencia K, van den Boorn JG, Denman CJ, Li M, Carlson JM, Hernandez C (2006). Therapeutic implications of autoimmune vitiligo T cells. Autoimmun. Rev..

[13] Steitz J, Wenzel J, Gaffal E, Tuting T (2004). Initiation and regulation of CD8+T cells recognizing melanocytic antigens in the epidermis: implications for the pathophysiology of vitiligo. Eur. J. Cell Biol..

[14] Maouia A, Sormani L, Youssef M, Helal AN, Kassab A, Passeron T (2017). Differential expression of CXCL9, CXCL10, and IFN-gamma in vitiligo and alopecia areata patients. Pigment Cell Melanoma Res..

[15] Rashighi M, Agarwal P, Richmond JM, Harris TH, Dresser K, Su MW (2014). CXCL10 is critical for the progression and maintenance of depigmentation in a mouse model of vitiligo. Sci. Transl. Med..

[16] Speeckaert R, Speeckaert M, De Schepper S, van Geel N (2017). Biomarkers of disease activity in vitiligo: a systematic review. Autoimmun. Rev..

[17] Strassner JP, Rashighi M, Ahmed Refat M, Richmond JM, Harris JE (2017). Suction blistering the lesional skin of vitiligo patients reveals useful biomarkers of disease activity. J. Am. Acad. Dermatol..

[18] Wang XX, Wang QQ, Wu JQ, Jiang M, Chen L, Zhang CF (2016). Increased expression of CXCR3 and its ligands in patients with vitiligo and CXCL10 as a potential clinical marker for vitiligo. Br. J. Dermatol..

[19] Ferrari SM, Fallahi P, Santaguida G, Virili C, Ruffilli I, Ragusa F (2017). Circulating CXCL10 is increased in non-segmental vitiligo, in presence or absence of autoimmune thyroiditis. Autoimmun. Rev..

[20] Groom JR, Luster AD (2011). CXCR3 ligands: redundant, collaborative and antagonistic functions. Immunol. Cell Biol..

[21] Harris JE, Harris TH, Weninger W, Wherry EJ, Hunter CA, Turka LA (2012). A mouse model of vitiligo with focused epidermal depigmentation requires IFN-gamma for autoreactive CD8(+) T-cell accumulation in the skin. J. Invest. Dermatol..

[22] Rashighi M, Harris JE (2015). Interfering with the IFN-gamma/CXCL10 pathway to develop new targeted treatments for vitiligo. Ann. Transl. Med..

[23] Abdallah M, El-Mofty M, Anbar T, Rasheed H, Esmat S, Al-Tawdy A (2018). CXCL-10 and Interleukin-6 are reliable serum markers for vitiligo activity: a multicenter cross-sectional study. Pigment Cell Melanoma Res..

[24] Rosmarin D, Pandya AG, Lebwohl M, Grimes P, Hamzavi I, Gottlieb AB (2020). Ruxolitinib cream for treatment of vitiligo: a randomised, controlled, phase 2 trial. Lancet.

[25] Rothstein B, Joshipura D, Saraiya A, Abdat R, Ashkar H, Turkowski Y (2017). Treatment of vitiligo with the topical Janus kinase inhibitor ruxolitinib. J. Am. Acad. Dermatol..

[26] Chen YF, Yang PY, Hu DN, Kuo FS, Hung CS, Hung CM (2004). Treatment of vitiligo by transplantation of cultured pure melanocyte suspension: analysis of 120 cases. J. Am. Acad. Dermatol..

[27] Lerner AB, Halaban R, Klaus SN, Moellmann GE (1987). Transplantation of human melanocytes. J. Invest. Dermatol..

[28] Olsson MJ, Juhlin L (1993). Repigmentation of vitiligo by transplantation of cultured autologous melanocytes. Acta Derm. Venereol..

[29] Redondo P, del Olmo J, Garcia-Guzman M, Guembe L, Prosper F (2008). Repigmentation of vitiligo by transplantation of autologous melanocyte cells cultured on amniotic membrane. Br. J. Dermatol..

[30] Shi YL, Weiland M, Li J, Hamzavi I, Henderson M, Huggins RH (2013). MicroRNA expression profiling identifies potential serum biomarkers for non-segmental vitiligo. Pigment Cell Melanoma Res..

[31] Richmond JM, Bangari DS, Essien KI, Currimbhoy SD, Groom JR, Pandya AG (2017). Keratinocyte-derived chemokines orchestrate t-cell positioning in the epidermis during vitiligo and may serve as biomarkers of disease. J. Invest. Dermatol..

[32] Kuo P, Tuong ZK, Teoh SM, Frazer IH, Mattarollo SR, Leggatt GR (2018). HPV16E7-induced hyperplasia promotes CXCL9/10 expression and induces CXCR3(+) T-cell migration to skin. J. Invest. Dermatol..

[33] Kastenmuller W, Brandes M, Wang Z, Herz J, Egen JG, Germain RN (2013). Peripheral prepositioning and local CXCL9 chemokine-mediated guidance orchestrate rapid memory CD8+ T cell responses in the lymph node. Immunity.

[34] Huang C, Li P, Wang B, Deng Y, Li J, Mao M (2020). Multi-factors associated with efficacy and adverse events of fractional erbium:YAG laser-assisted delivery of topical betamethasone for stable vitiligo: a retrospective analysis. Lasers Surg. Med..

[35] Ibrahim ZA, Hassan GF, Elgendy HY, Al-Shenawy HA (2019). Evaluation of the efficacy of transdermal drug delivery of calcipotriol plus betamethasone versus tacrolimus in the treatment of vitiligo. J. Cosmet. Dermatol..

[36] Li J, Fu WW, Zheng ZZ, Zhang QQ, Xu Y, Fang L (2011). Suction blister epidermal grafting using a modified suction method in the treatment of stable vitiligo: a retrospective study. Dermatol. Surg. Off. Publ. Am. Soc. Dermatol. Surg..

[37] Kim HU, Yun SK (2000). Suction device for epidermal grafting in vitiligo: employing a syringe and a manometer to provide an adequate negative pressure. Dermatol. Surg. Off. Publ. Am. Soc. Dermatol. Surg..

